# What Went Wrong with the IMMUNI Contact-Tracing App in Italy? A Cross-Sectional Survey on the Attitudes and Experiences among Healthcare University Students

**DOI:** 10.3390/life12060871

**Published:** 2022-06-10

**Authors:** Claudia Isonne, Maria Roberta De Blasiis, Federica Turatto, Elena Mazzalai, Carolina Marzuillo, Corrado De Vito, Paolo Villari, Valentina Baccolini

**Affiliations:** Department of Public Health and Infectious Diseases, Sapienza University of Rome, 00185 Rome, Italy; mariaroberta.deblasiis@uniroma1.it (M.R.D.B.); federica.turatto@uniroma1.it (F.T.); elena.mazzalai@uniroma1.it (E.M.); carolina.marzuillo@uniroma1.it (C.M.); corrado.devito@uniroma1.it (C.D.V.); paolo.villari@uniroma1.it (P.V.); valentina.baccolini@uniroma1.it (V.B.)

**Keywords:** digital contact tracing, IMMUNI app, COVID-19, students

## Abstract

The adoption of digital contact-tracing apps to limit the spread of SARS-CoV-2 has been sup-optimal, but studies that clearly identify factors associated with the app uptake are still limited. In April 2021, we administered a questionnaire to healthcare university students to investigate their attitudes towards and experiences of the IMMUNI app. A multivariable logistic regression model was built to identify app download predictors. Adjusted odds ratios (aORs) and 95% confidence intervals (CIs) were calculated. We surveyed 247 students. Most respondents (65.6%) had not downloaded IMMUNI, reporting as the main reason the perceived app uselessness (32.7%). In the multivariable analysis, being advised to use the app (aOR: 3.21, 95%CI: 1.80–5.73), greater fear of infecting others (aOR: 1.50, 95%CI: 1.01–2.23), and greater trust in the institutional response to the emergency (aOR: 1.33, 95%CI: 1.00–1.76) were positively associated with the outcome, whereas greater belief in the “lab-leak theory” of COVID-19 was a negative predictor (aOR: 0.75, 95%CI: 0.60–0.93). Major technical issues were reported by app users. Targeted strategies aimed at improving awareness of digital health applications should be devised. Furthermore, institutions should invest in the development of these technologies, to minimize technical issues and make them accessible to the entire population.

## 1. Introduction

Contact tracing has long been a key public health tool for slowing or stopping the spread of infectious diseases [1]. It allows rapid and accurate identification of individuals who have been exposed to confirmed or probable cases (contacts) and, thus, the infection’s chain of transmission to be broken [2]. During the COVID-19 pandemic, contact tracing has assumed a critical role in mitigating transmission of the SARS-CoV-2 virus and limiting its dramatic effects on health systems and societies [3,4,5]. Nevertheless, several challenges in using the traditional contact-tracing strategy have become apparent in many countries [6]. Among these, the scarcity of previously trained personnel, the short time between infection and the onset of symptoms, as well as a possible recall bias, may have hindered the effectiveness of this surveillance system [7]. For these reasons, and because a number of digital health technologies have been implemented successfully in recent years, several mobile applications have been developed to support the traditional approach by enabling digital contact tracing [8,9].

Using Bluetooth or GPS technology together with an appropriate app, it is possible to geolocate and record every device that has been in close proximity with another [10]. This allows users to be tracked and notified when they have been near the device of a person who reports testing positive for SARS-CoV-2, and, consequently, to take preventive measures, such as quarantine [11]. Recognizing the public health potential of digital contact tracing (DCT) tools, which is underpinned by modelling studies that demonstrate how DCT could help control the spread of SARS-CoV-2 [8], several countries have introduced these systems, some of them with positive experiences, especially Eastern countries [7]. In Italy, the DCT app “IMMUNI” was launched in June 2020 and its use was on voluntary basis [12]. Briefly, when installed on a smartphone, IMMUNI emits a Bluetooth signal that includes a random code. It does this on a continuous basis. When a person approaches another one, their smartphones exchange these codes and store them in their memory, thus making note of that contact. When a person is notified of testing positive to SARS-CoV-2, with the help of healthcare personnel, the user is able to report this result to IMMUNI, sharing his or her random codes and alerting the people he or she has been in close contact with [13]. However, despite initial interest in this innovation in Italy, and in similar apps in other Western countries, the intense international debate over the ethical, legal, and societal implications has hampered efforts to implement DCT strategies [8,14,15].

Recent evidence shows a generally positive attitude towards DCT apps [16], but issues of cyber security, variable risk perception, and poor awareness of benefits have been indicated as barriers to their use [17,18]. However, studies that clearly identify factors associated with app uptake are still limited, and results are mixed [19,20,21]. Therefore, it is critical to further investigate the factors that may have hindered app use. This is especially pertinent among young people, who on one hand are at higher risk of SARS-CoV-2 infection given their frequent opportunities to socialize [22], while on the other hand they have a greater degree of digital literacy and acceptance of downloaded apps [21]. The objective of our study was to investigate the attitude towards the IMMUNI app in a sample of healthcare university students. We also explored their experiences of using it as well as the main barriers to its download. Specifically, we aimed to identify the key factors associated with its uptake among a cohort of people who have been trained to adopt health prevention behavior, and, thereby, to better understand what may have hampered its adoption in a population that should be receptive to DCT.

## 2. Materials and Methods

### 2.1. Setting and Participants

This cross-sectional study was conducted at Sapienza University of Rome between 14 and 19 April 2021. An online survey was administered to students enrolled in the healthcare area (i.e., three nursing science courses and one physiotherapy course). Access to the questionnaire was via a link sent by e-mail to the students’ institutional e-mail addresses. The study was performed in accordance with the World Medical Association Declaration of Helsinki. Participants were asked for their consent and were guaranteed anonymity in the information collected. The institutional ethics board of the Umberto I teaching hospital/Sapienza University of Rome approved this study (protocol number 571/2021).

### 2.2. Questionnaire

The questionnaire was self-administered and took approximately five minutes to fill out (Supplementary Materials). It consisted of a maximum of 33 closed-ended questions grouped into three sections.

The first section aimed to collect sociodemographic information: age, gender, field of study, year of study, nationality, Italian Region, finances (i.e., with the financial resources at your disposal, how well do you get to the end of the month?), main source of health information (i.e., what is your main source of health information?), health literacy (HL) (i.e., how often do you need to have someone help when you read instructions, pamphlets, or other written material from your doctor or pharmacy? [23]), chronic pathologies, and the occurrence and symptoms of a SARS-CoV-2 infection in the past.

The second section explored students’ perceptions of and attitudes towards the COVID-19 pandemic. Specifically, we asked them to rate on a 5-point scale (from 1 [very low] to 5 [very high]) how great was their fear of getting the SARS-CoV-2 infection, fear of infecting others, and their concern about the COVID-19 emergency. We also asked students to express their feelings in relation to the pandemic (i.e., depression, anxiety, and anger, from 1 [not at all] to 5 [extremely]), to self-report adherence to COVID-19 precautionary measures (i.e., compliance with social distancing and use of mask, from 1 [not at all] to 5 [extremely]), their trust in institutions (i.e., on a scale from 1 [not at all] to 5 [extremely]; how much do you trust the response of the institutions to the emergency?), and their belief that the virus originated from a laboratory in Wuhan (i.e., on a scale from 1 [not at all] to 5 [extremely]; how much do you believe in the “lab-leak theory” of the origin of COVID-19?). Finally, we asked whether someone had advised them to download and use IMMUNI, when they had actually downloaded it and whether they were still using it.

The third section was different for students who had downloaded the app and those who had not. In the first group, we investigated the main reasons for such a download and their assessment of some app features (i.e., on a scale from 1 [very poor] to 5 [excellent], how would you assess the privacy features, ease of use, usefulness, and intuitiveness?). In addition, students were asked to report their personal experience with app notifications. Two possible scenarios were investigated: (i) receipt of at least one notification as a potential contact, and the nature of their post-notification behavior, or (ii) at least one notification via the app of having a SARS-CoV-2 infection, and their assessment of the notification process (from very lacking to very good) together with the difficulties encountered in submitting the notification, if applicable. For the students reported to have not download the app, the third section explored their attitudes. We asked the main reason why they did not download IMMUNI, and to rate on a scale from 1 (not at all) to 5 (definitely) how effective some hypothetical incentives would have been in promoting app uptake: (i) receiving concrete feedback on how the app could help limit the virus spread; (ii) being informed about the app’s uptake among the population; (iii) making app download mandatory; (iv) having the opportunity to give feedback on the technical aspects of the app; (v) receiving more information about personal data collection and management; and (vi) receiving an economic reward.

### 2.3. Statistical Analysis

Descriptive statistics were obtained using median and interquartile range, or mean and standard deviation, for continuous variables and proportions for dichotomous and categorical variables. Student age was dichotomized using 21 years as a cut-off. Participants were classified into four groups according to their year of study: first-, second-, or third-year students, and students outside prescribed courses. As for nationality, respondents were classed as Italian or non-Italian. Health literacy was categorized into two groups: adequate HL (answering never/rarely) and non-adequate HL (answering sometimes/often/always) [24]. Chronic pathologies were grouped into nine categories: none, autoimmune disease, cardiovascular disease, endocrine disease, genetic disease, gynecological disease, psychiatric disease, respiratory disease, and cancer. SARS-CoV-2 infection was categorized into four groups: no infection, asymptomatic, mild symptoms, and moderate/severe symptoms.

For the univariable analysis, the Mann–Whitney U test was used to compare continuous variables between students who had download IMMUNI and the students who had not, whereas Pearson’s chi-squared test or Fisher’s test was used for dichotomous and categorical variables, as appropriate. A multivariable logistic regression model was built to identify predictors of app download. Variables were included in the model based on expert opinion. Multicollinearity was checked using as threshold a variance inflation factor of 5. The Hosmer and Lemeshow test was used to evaluate the goodness of fit of the model. As a result, the final model consisted of the following variables: age (<21 vs. ≥21 years), gender (male vs. female), HL (inadequate vs. adequate), fear of getting the SARS-CoV-2 infection (continuous), fear of infecting others (continuous), concern about the COVID-19 pandemic (continuous), trust in the response of the institutions to the emergency (continuous), belief in the “lab-leak theory” of the origin of COVID-19 (continuous), and receipt of some advice to download the app (yes vs. no). Adjusted odds ratios (ORs) and 95% confidence intervals (CIs) were calculated.

All analyses were performed using Stata (StataCorp LLC, 4905 Lakeway Drive, College Station, TX, USA), version 17.0. A two-sided *p*-value < 0.05 was considered statistically significant.

## 3. Results

A total of 247 students answered the questionnaire (response rate: 72.4%). Of the 85 students who had downloaded IMMUNI (34.4%), more than half had done it immediately on launch of the app (N = 48), and the remaining participants between September and November 2020, but all of them were still using it in April 2021 [Table 1]. The two groups were of a similar age. Most were females (71.8% vs. 77.2%); almost three in every four attended undergraduate nursing courses (68.2% vs. 75.3%) and more than 90% were enrolled as first- or second- year students. Only a minority of responders were non-Italian (around 2.5%) and approximately half the Italian respondents came from the Lazio Region. More than 60% of the students in both groups reported that they got to the end of the month (financially) very well or well enough. The mass media was indicated as the main source of health information in both groups (around 40%), followed by social networks and the Internet, whereas only a limited number of students reported not looking for any health information (~1%). Most students showed adequate HL, with more than two thirds answering that they never or rarely needed help understanding medical material. The vast majority of respondents in both groups did not suffer from any chronic conditions and had never contracted the SARS-CoV-2 infection.

No significant difference in terms of fear of contracting SARS-CoV-2 was observed between those who downloaded the app compared to those who did not [Table 2]. By contrast, although it did not reach statistical significance (mean score: 4.5 vs. 4.2), the first cohort seemed to have a slightly greater fear of infecting others. Concern about the COVID-19 pandemic did not differ (mean score: 3.9 vs. 3.8), and neither did the students’ feelings in relation to the pandemic, among which, depression was the most reported in both groups (depression, mean score: 3.1 vs. 3.2; anxiety, mean score: 2.9 vs. 3.0; anger, mean score: 2.5 vs. 2.8). Self-reported adherence to COVID-19 precautionary measures (i.e., respect of social distancing and use of mask) was slightly higher in the first group, albeit not significantly (mean score: 4.8 vs. 4.6 in both items). Conversely, the group that downloaded the app had a greater trust in the response of the institutions to the emergency (mean score: 3.6 vs. 3.3). By contrast, students that did not download the app had a significantly greater belief that the virus originated from a laboratory (mean score: 2.4 vs. 1.9). Finally, a greater proportion of students among those who had downloaded the app reported they had been advised to do so (64.7% vs. 38.3%).

The main reasons for uptake of the app, among those who downloaded it, were sense of duty (40.0%) and respect for others (30.6%), followed by fear of getting the infection (20.0%), and curiosity (9.4%) [Table 3]. On average, students rated as very good the privacy features of the app (mean score: 4.0), and they found it easy to use (mean score: 3.8), but also quite intuitive and useful (mean score: 3.4 for both). Overall, only 8.2% of the students who downloaded the app received at least one alert that they were a potential contact and most followed the app advice (around 70%). Similarly, only seven students (8.2%) tried to notify a positive COVID-19 test through the app, but most of them were not successful (71.4%). Of these, one student was unable to get the National Unique Code (CUN) whereas three participants were unable to enter the CUN in the app. Almost three quarters of these students rated the notification process as lacking or very lacking (71.4%).

Students who did not download IMMUNI reported that the main reason for not doing so was the belief that it was useless (32.7%) and because they did not know they had to do it (23.5%), but also for technical issues (almost 20%) and, albeit less frequently, because of a distrust in data management (around 16%) [Table 4]. In addition, a small percentage reported hearing of negative experiences (5.6%). As for the hypothetical incentives that could increase app uptake, information on how app usage could impact virus transmission dynamics was the main driver (mean score: 3.5), followed by information on the app’s uptake among the population (mean score: 3.4) and making its download mandatory (mean score: 3.4). A slightly lower importance was attributed to the opportunity to give feedback on the technical aspects of the app (mean score: 3.2) and information about personal data collection and management (mean score: 3.1). Lastly, having an economic reward seemed to be the least effective incentive (mean score: 2.4).

In the multivariable analysis [Table 5], participants who had received some advice to download the app seemed to have the highest odds of IMMUNI uptake (aOR: 3.21, 95% CI: 1.80–5.73). Similarly, reporting a higher fear of infecting other people was associated with higher likelihood of app download (aOR: 1.50, 95% CI: 1.01–2.23), as well as a greater trust in the response of the institutions to the emergency (aOR: 1.33, 95% CI: 1.00–1.76). On the other hand, greater belief in the “lab-leak theory” of the origin of COVID-19 was negatively associated with download (aOR: 0.75, 95% CI: 0.60–0.93). By contrast, age, gender, HL, fear of getting the SARS-CoV-2 infection, and concern about the COVID-19 pandemic did not seem to be predictors of the outcome.

## 4. Discussion

The usefulness of DCT apps has been a subject of intense discussion during the COVID-19 pandemic [18,25]. Most governments have struggled with low participation rates, which, in turn, have limited the effectiveness of these tools, contributing to the idea that they are useless and, thus, hindering their adoption [21]. Recently, several researchers have investigated the acceptability and use of contact tracing apps. Most studies are based on surveys assessing the uptake of DCT apps among different population subgroups with a focus on hypothetical tools or the intention to use it, but only a few collect information on the use of an existing app [21]. The majority of documents report the real uptake using data of national statistics without a scientific and theoretical background, while other studies are critical viewpoints arguing on the ethical, technical, political, and scientific impact of contact tracing apps on society [21]. In our study, we found a higher uptake rate of the IMMUNI app compared to that in the general Italian population [12], probably because our sample consisted of students attending healthcare courses, who are more likely to be committed to health prevention strategies [26]. In addition, the fact that, in our analysis, almost all students had the opportunity to download the app since they owned a smartphone, in contrast to the official data where it is more difficult to estimate the number of people eligible for the app uptake, may have contributed to such discrepancy [18]. Nevertheless, we found that use of the DCT app was relatively limited, albeit at a similar rate to uptake of comparable apps in other European nations [21]. This is a concern, however, as these students are the healthcare workforce of tomorrow, and, therefore, it is imperative to implement educational programs that further encourage the adoption of preventive strategies [27]. Moreover, it should be mentioned that the current increase in virus transmission rates due to the omicron variant, and the concomitant abolition of restrictive measures at both the national and regional level, could make it difficult to promptly identify the transmission chain using traditional methods [28]. In this scenario, a high uptake rate of the app would have some advantages, including the support to trace contacts, but also would make the population autonomous in the timely application of the preventive measures and create an environment in which citizens are effectively engaged in maintaining their personal and community health [29].

As for determinants, IMMUNI uptake was not associated with any socio-demographic characteristic, including HL, probably because of the healthcare curricula of our students, but some attitudes towards the pandemic seemed slightly different between the two groups. Risk perception was confirmed to be a key driver, but it changed over time; thus, people may have become used to a high level of risk as the pandemic continued, consequently reducing their motivation to act and use DCT tools [18]. Such changes in risk perception could explain the app download trend in Italy, which consisted of an initial peak when the app was launched in June 2020 (up to 600,000 downloads in a single day), followed by another massive increase in downloads at the beginning of the second wave, reaching more than 200,000 per day. This then tailed off to around 2000 downloads per day until April 2021, when the number of cases was limited and the vaccination campaign was at full deployment [30]. Among other factors explored, a few studies have already documented how high levels of trust in governments and health authorities can motivate people to adhere to prevention strategies [18,19,31]. It is fundamental that institutions convey official messages clearly and coherently, and combating disinformation from other sources as much as possible [18]. In addition, good communication seems important for increasing the acceptability of the app in our study population: the strongest predictor of app uptake in our analyses was being advised to download it, while a reason for non-adoption was a lack of awareness of the app. Lastly, our participants belonged to an age group that may be characterized by sociability, the importance of self-identifying with a peer group and the influence of peers on the adoption of health behavior [32]; therefore, exploiting these social mechanisms by implementing targeted communication strategies is likely to be effective at reaching a large fraction of this population [33].

As aforementioned, at the time of the survey (April 2021), Italy was at the end of the second wave, which had been characterized by a high incidence of SARS-CoV-2 infections during the fall and winter of 2020–2021 [34]. Hence, in the low transmission risk scenario of April 2021, it was not unexpected that we found a low perception of the utility of the DCT tool, similarly to other studies [19,21]. Within this context, communication policies that help people understand the importance of such measures in safeguarding their own and community health should be devised [33]. Such campaigns are most effective when risk perception is high, because people are motivated to take action to protect themselves, which potentiates DCT acceptance [18]. In fact, it is well known that a low adoption rate is the main barrier to the effectiveness of these apps [35] and the poor uptake may be responsible for the limited number of app notifications that our students reported. However, while potential contacts mostly followed the health recommendations provided by the app, which is encouraging because it highlights their awareness of the need to adopt preventive measures promptly, a substantial proportion of our students claimed they were hampered by technical issues with the notification process. This underlines the importance of investing in technical improvements of these apps and making them easy to use for the entire population, most of whom are less digitally literate than young people [35,36]. It is, in fact, important to highlight that several technical skill challenges could occur, such as some people not knowing how to download and install an app, or how to interact with it, thus limiting its acceptance and usage [18].

Interestingly, our findings contrast with a few international studies that report how concerns about data privacy can negatively impact DCT app adoption [16,31,37,38]. This could be explained by the fact that, compared to other, similar apps, IMMUNI collects relatively few data [39]. Additionally, our cohort was composed of university students, who may be accustomed to sharing their data on the web and not be particularly concerned about privacy issues [40]. Lastly, as for incentives that might promote the adoption of DCT apps, despite their recognized importance [41], few studies have investigated this aspect and available evidence focuses only on financial incentives [42,43]. In our study, it was the app’s actual utility (or otherwise) that seemed to influence its adoption rate. This highlights how the feeling of being engaged may motivate people to participate in a DCT system and confirms the importance of investing in communication policies that point out the potential health benefits of using such technologies [44].

This study has some limitations. Firstly, the cross-sectional design hindered the opportunity to draw causal conclusions between app uptake and associated factors. Secondly, the relatively low number of participants might have limited the statistical power. Thirdly, since we investigated students enrolled in healthcare degree courses, results are not generalizable to all university students. For these reasons, further analyses should be conducted comparing students of both medical and non-medical subjects to highlight possible differences between the two groups. However, to the best of our knowledge, this is the first study that investigates how Italian students relate to IMMUNI by analyzing factors that affect its adoption. Since these factors are specific and different across population subgroups, it is fundamental to assess and monitor them over time, so that they can be addressed in the development of similar technologies. In addition, we were able to examine the experience of students that used the app and also to explore possible incentives to encourage reluctant or disinterested users. The data provided in this study may support policymakers in developing effective strategies for the promotion of app uptake and, more broadly, to facilitate engagement of people with digital health prevention measures.

## 5. Conclusions

The results of our study suggest that more efforts should be made aimed at raising population awareness on the usefulness of health digital technologies, restoring their confidence in health authorities, and limiting the spread of disinformation. To maximize the active engagement of the population, stakeholders should implement strategies that provide quality, clear, targeted, and straightforward information. Furthermore, institutions should invest in the development of these technologies, minimizing technical issues and facilitating their use in the population. Since university students represent an amenable target audience, because they are undergoing (often relevant) training and are, therefore, particularly receptive to educational campaigns, interventions should focus on improving their knowledge and awareness of how adhering to these strategies can contribute to safeguarding individual and public health.

## Figures and Tables

**Table 1:** Students’ sociodemographic characteristics vs. IMMUNI app download. Results are expressed as frequency (percentage).

	App Download		
	Yes (N = 85)	No (N = 162)	*p*-Value *
Age			0.463
<21 years	43 (50.6)	74 (45.6)	
≥21 years	42 (49.4)	88 (54.3)	
Gender			0.350
Female	61 (71.8)	125 (77.2)	
Male	24 (28.2)	37 (22.8)	
Field of study			0.235
Nursing science	58 (68.2)	122 (75.3)	
Physiotherapy	27 (31.8)	40 (24.7)	
Year of study			0.911
First	37 (43.5)	69 (42.6)	
Second	39 (45.9)	79 (48.8)	
Third	8 (9.4)	13 (8.0)	
Outside prescribed course	1 (1.2)	1 (0.6)	
Nationality			0.999
Italian	83 (97.6)	158 (97.5)	
Non-Italian	2 (2.4)	4 (2.5)	
Italian Region (N = 241)			0.049
Abruzzo	0 (0.0)	2 (1.3)	
Calabria	3 (3.6)	5 (3.1)	
Campania	3 (3.6)	11 (6.9)	
Lazio	41 (49.4)	90 (56.9)	
Puglia	18 (21.7)	13 (8.2)	
Sardegna	1 (1.2)	0 (0.0)	
Sicilia	17 (20.5)	29 (18.4)	
Umbria	0 (0.0)	1 (0.6)	
Veneto	1 (0.0)	2 (1.3)	
Missing	2 (0.0)	5 (3.1)	
Finances			0.169
I have many difficulties	6 (7.0)	11 (6.8)	
I have some difficulties	26 (30.6)	48 (29.6)	
Managing well enough	34 (40.0)	83 (51.2)	
Managing very well	19 (22.4)	20 (12.4)	
Main source of health information			0.999
Mass media	35 (41.2)	65 (40.1)	
Web	20 (23.5)	39 (24.1)	
Social network	29 (34.1)	56 (34.6)	
None	1 (1.2)	2 (1.2)	
Health literacy			0.360
Non-adequate	24 (28.2)	55 (33.9)	
Adequate	61 (71.8)	107 (66.0)	
Chronic pathologies			0.164
None	72 (84.7)	149 (91.9)	
Autoimmune disease	3 (3.5)	1 (0.6)	
Cardiovascular disease	0 (0.0)	2 (1.2)	
Cancer	1 (1.2)	0 (0.0)	
Endocrine disease	1 (1.2)	2 (1.2)	
Genetic disease	1 (1.2)	1 (0.6)	
Gynecological disease	1 (1.2)	0 (0.0)	
Psychiatric disease	0 (0.0)	1 (0.6)	
Respiratory disease	6 (7.1)	6 (3.7)	
SARS-CoV-2 infection			0.865
No infection	78 (91.7)	149 (92.0)	
Asymptomatic	1 (1.2)	1 (0.6)	
Mild symptoms	5 (5.9)	8 (4.9)	
Moderate/severe symptoms	1 (1.2)	4 (2.5)	

* Pearson’s chi-squared test or Fisher test.

**Table 2:** Students’ perceptions of and attitudes towards SARS-CoV-2 pandemic vs. IMMUNI app download. Results are expressed as mean (standard deviation) or frequency (percentage).

	App Download		
	Yes (N = 85)	No (N = 162)	*p*-Value *
Fear of getting the SARS-CoV-2 infection	2.8 (1.1)	2.7 (1.2)	0.545
Fear of infecting others	4.5 (0.8)	4.2 (1.1)	0.051
Concern about the COVID-19 emergency	3.9 (1.0)	3.8 (1.1)	0.954
Feelings about the COVID-19 pandemic			
Depression	3.1 (1.3)	3.2 (1.3)	0.578
Anxiety	2.9 (1.3)	3.0 (1.4)	0.547
Anger	2.5 (1.4)	2.8 (1.4)	0.122
Adherence to COVID-19 precautionary measures			
Maintaining physical distance	4.8 (0.6)	4.6 (0.7)	0.074
Use of mask	4.8 (0.5)	4.6 (0.7)	0.137
Trust in institutional response to the emergency	3.6 (1.0)	3.3 (1.1)	0.025
Belief in the lab-leak theory of COVID-19 origin	1.9 (1.3)	2.4 (1.4)	0.003
Receipt of advice to download the app			<0.001
No	30 (35.3)	100 (61.7)	
Yes	55 (64.7)	62 (38.3)	

COVID-19: coronavirus diseases 2019. * Pearson’s chi-squared test for categorical variables and Mann–Whitney U test for continuous variables.

**Table 3:** Attitudes and experiences of surveyed students who downloaded the IMMUNI App. Results are expressed as mean (standard deviation) or frequency (percentage).

	N = 85
Main reason for the app download	
Sense of duty	34 (40.0)
Respect for others	26 (30.6)
Fear of getting the infection	17 (20.0)
Curiosity	8 (9.4)
Assessment of app features	
Privacy	4.0 (1.1)
Ease of use	3.8 (1.1)
Usefulness	3.4 (1.3)
Intuitiveness	3.4 (1.3)
Receipt of at least one contact notification	
No	78 (91.8)
Yes	7 (8.2)
Post-notification behavior (N = 7)	
I received and followed the advice provided by the app	5 (71.4)
I received the advice, but I did not do anything	2 (28.6)
Notification of positivity (N = 7)	
No, I was not able to	5 (71.4)
Yes, I was given the CUN, and I entered the requested data on the app	1 (14.3)
Yes, I provided the CUN to the healthcare professional who contacted me for contact-tracing purposes	1 (14.3)
Assessment of the notification process (N = 7)	
Very lacking	4 (57.1)
Lacking	1 (14.3)
Good	1 (14.3)
Very good	1 (14.3)
Challenges/technical issues (N = 7)	
I was unable to get the CUN	1 (14.3)
I was unable to enter the CUN in the app even after calling the IMMUNI call center	1 (14.3)
I was unable to enter the CUN in the app and I did not know that I could call the IMMUNI call center	2 (28.6)
I did not had any difficulty	2 (28.6)
Missing	1 (14.3)

CUN: National Unique Code.

**Table 4:** Attitudes of surveyed students who did not download the IMMUNI App. Results are expressed as mean (standard deviation) or frequency (percentage).

	N = 162
Reason for not downloading the app	
I do not think it is useful	53 (32.7)
I did not know I had to download the app	38 (23.5)
Technical problems (e.g., no smartphone, operating system incompatibility, battery problems, insufficient storage on the phone, etc.)	31 (19.1)
I do not trust data management (privacy issue)	26 (16.1)
I have heard of negative personal experiences	9 (5.6)
Other reasons	5 (3.1)
Effectiveness of hypothetical incentives in increasing the app uptake	
Information on how usage can impact transmission dynamics	3.5 (1.3)
Information on the app’s uptake among the population	3.4 (1.3)
Making the app download mandatory	3.4 (1.4)
Opportunity to give feedback on the technical aspects of the app	3.2 (1.4)
Information about personal data collection and management	3.1 (1.4)
Economic reward	2.4 (1.5)

**Table 5:** Multivariable logistic regression model for IMMUNI app download among the students surveyed between 14 and 19 April 2021, Sapienza University of Rome.

	App Download	
	aOR (95% CI)	*p*-Value
Age		
<21 years	Ref.	
≥21 years	0.77 (0.43–1.27)	0.373
Gender		
Female	Ref.	
Male	1.48 (0.75–2.89)	0.265
Health literacy		
Adequate	Ref.	
Non-adequate	0.69 (0.36–1.30)	0.256
Fear of getting the SARS-CoV-2 infection	1.04 (0.79–1.37)	0.776
Fear of infecting others	1.50 (1.01–2.23)	0.042
Concern about the COVID-19 emergency	0.85 (0.62–1.17)	0.327
Trust in institutional response to the emergency	1.33 (1.00–1.76)	0.049
Belief in lab-leak theory of COVID-19 origin	0.75 (0.60–0.93)	0.011
Receipt of advice to download the app		
No	Ref.	
Yes	3.21 (1.80–5.73)	<0.001

aOR: adjusted Odds Ratio. CI: confidence interval. COVID-19: coronavirus diseases 2019.

## Data Availability

The data presented in this study are available on request from the corresponding author.

## Supplementary Materials

The following supporting information can be downloaded at: https://www.mdpi.com/article/10.3390/life12060871/s1, Survey questionnaire.

## References

[1] Hossain A.D., Jarolimova J., Elnaiem A., Huang C.X., Richterman A., Ivers L.C. (2022). Effectiveness of contact tracing in the control of infectious diseases: A systematic review. Lancet Public Health.

[2] Shelby T., Schenck C., Weeks B., Goodwin J., Hennein R., Zhou X., Spiegelman D., Grau L.E., Niccolai L., Bond M. (2021). Lessons Learned From COVID-19 Contact Tracing During a Public Health Emergency: A Prospective Implementation Study. Front. Public Health.

[3] World Health Organization (WHO) Regional Office for Europe (2020). Contact tracing in the context of COVID-19. Interim guidance. Pediatr. Med. Rodz..

[4] Mazza C., Girardi D., Gentile L., Gaeta M., Signorelli C., Odone A. (2021). Public health effectiveness of digital contact tracing in the COVID-19 pandemic: A systematic review of available data. Acta Biomed..

[5] European Centre for Disease Prevention and Control (ECDC) (2022). Analysis of COVID-19 Contact Tracing Data from Ireland, Italy and Spain—2020 Data.

[6] Anglemyer A. (2020). Digital contact tracing technologies in epiDemics: A rapid review. Saudi Med. J..

[7] O’Connell J., O’Keeffe D.T. (2021). Contact Tracing for Covid-19—A Digital Inoculation against Future Pandemics. N. Engl. J. Med..

[8] Blasimme A., Ferretti A., Vayena E. (2021). Digital Contact Tracing Against COVID-19 in Europe: Current Features and Ongoing Developments. Front. Digit. Health.

[9] World Health Organization, European Centre for Disease Prevention and Control (2021). Indicator Framework to Evaluate the Public Health Effectiveness of Digital Proximity Tracing Solutions.

[10] Zeng K., Bernardo S.N., Havins W.E. (2020). The use of digital tools to mitigate the COVID-19 pandemic: Comparative retrospective study of six countries. JMIR Public Health Surveill..

[11] Hernández-Quevedo C., Scarpetti G., Webb E. How Do Countries Structure Contact Tracing Operations and What Is the Role of Apps?. https://analysis.covid19healthsystem.org/index.php/2020/06/18/how-do-countries-structure-contact-tracing-operations-and-what-is-the-role-of-apps/.

[12] Scrivano N., Gulino R.A., Giansanti D. (2022). Digital Contact Tracing and COVID-19: Design, Deployment, and Current Use in Italy. Healthcare.

[13] Ministero Della Salute IMMUNI—Hai Qualche Domanda?. https://www.immuni.italia.it/faq.html.

[14] World Health Organization (2020). Ethical Considerations to Guide the Use of Digital Proximity Tracking Technologies for COVID-19 Contact Tracing.

[15] Ranisch R., Nijsingh N., Ballantyne A., van Bergen A., Buyx A., Friedrich O., Hendl T., Marckmann G., Munthe C., Wild V. (2021). Digital contact tracing and exposure notification: Ethical guidance for trustworthy pandemic management. Ethics Inf. Technol..

[16] Altmann S., Milsom L., Zillessen H., Blasone R., Gerdon F., Bach R., Kreuter F., Nosenzo D., Toussaert S., Abeler J. (2020). Acceptability of app-based contact tracing for COVID-19: Cross-country survey study. JMIR mHealth uHealth.

[17] Walrave M., Waeterloos C., Ponnet K. (2020). Adoption of a contact tracing app for containing COVID-19: A health belief model approach. JMIR Public Health Surveill..

[18] Chen A.T.-Y., Thio K.W. (2021). Exploring the drivers and barriers to uptake for digital contact tracing. Soc. Sci. Humanit. Open.

[19] Von Wyl V., Höglinger M., Sieber C., Kaufmann M., Moser A., Serra-Burriel M., Ballouz T., Menges D., Frei A., Puhan M.A. (2021). Drivers of acceptance of COVID-19 proximity tracing apps in Switzerland: Panel survey analysis. JMIR Public Health Surveill..

[20] Blom A.G., Wenz A., Cornesse C., Rettig T., Fikel M., Friedel S., Möhring K., Naumann E., Reifenscheid M., Krieger U. (2021). Barriers to the large-scale adoption of a COVID-19 contact tracing app in Germany: Survey study. J. Med. Internet Res..

[21] Montagni I., Roussel N., Thiébaut R., Tzourio C. (2021). Health care students’ knowledge of and attitudes, beliefs, and practices toward the french covid-19 app: Cross-sectional questionnaire study. J. Med. Internet Res..

[22] Baccolini V., Renzi E., Isonne C., Migliara G., Massimi A., De Vito C., Marzuillo C., Villari P. (2021). COVID-19 vaccine hesitancy among italian university students: A cross-sectional survey during the first months of the vaccination campaign. Vaccines.

[23] Bonaccorsi G., Grazzini M., Pieri L., Santomauro F., Ciancio M., Lorini C. (2017). Assessment of Health Literacy and validation of single-item literacy screener (SILS) in a sample of Italian people. Ann. Dell’istituto Super. Sanità.

[24] Baccolini V., Rosso A., Di Paolo C., Isonne C., Salerno C., Migliara G., Prencipe G., Massimi A., Marzuillo C., De Vito C. (2021). What is the Prevalence of Low Health Literacy in European Union Member States? A Systematic Review and Meta-analysis. J. Gen. Intern. Med..

[25] O’Connell J., Abbas M., Beecham S., Buckley J., Chochlov M., Fitzgerald B., Glynn L., Johnson K., Laffey J., McNicholas B. (2021). Best practice guidance for digital contact tracing apps: A cross-disciplinary review of the literature. JMIR mHealth uHealth.

[26] Tempski P., Arantes-Costa F.M., Kobayasi R., Siqueira M.A.M., Torsani M.B., Amaro B.Q.R.C., Nascimento M.E.F.M., Siqueira S.L., Santos I.S., Martins M.A. (2021). Medical students’ perceptions and motivations during the COVID-19 pandemic. PLoS ONE.

[27] Baccolini V., Isonne C., Salerno C., Giffi M., Migliara G., Mazzalai E., Turatto F., Sinopoli A., Rosso A., De Vito C. (2022). The association between adherence to cancer screening programs and health literacy: A systematic review and meta-analysis. Prev. Med..

[28] European Centre for Disease Prevention and Control (2022). Assessment of the further spread and potential impact of the SARS-CoV-2 Omicron variant of concern in the EU/EEA, 19th update Risk assessed. ECDC Stock..

[29] Megnin-Viggars O., Carter P., Melendez-Torres G.J., Weston D., Rubin G.J. (2020). Facilitators and barriers to engagement with contact tracing during infectious disease outbreaks: A rapid review of the evidence. PLoS ONE.

[30] Ministero della Salute I Numeri di IMMUNI. https://www.immuni.italia.it/dashboard.html.

[31] Jones K., Thompson R. (2021). To use or not to use a COVID-19 contact tracing app: Mixed methods survey in Wales. JMIR mHealth uHealth.

[32] Overbeek G., Bot S.M., Meeus W.H.J., Sentse M., Knibbe R.A., Engels R. (2011). Where it’s at! the role of best friends and peer group members in young adults’ alcohol use. J. Res. Adolesc..

[33] Shopova T. (2014). Digital literacy of students and its improvement at the university. J. Effic. Responsib. Educ. Sci..

[34] The COVID-19 Task Force of the Department of Infectious Diseases and the IT Service Istituto Superiore di Sanità COVID-19 Integrated Surveillance Data in Italy. https://www.epicentro.iss.it/coronavirus/sars-cov-2-dashboard.

[35] Akinbi A., Forshaw M., Blinkhorn V. (2021). Contact tracing apps for the COVID-19 pandemic: A systematic literature review of challenges and future directions for neo-liberal societies. Health Inf. Sci. Syst..

[36] Leslie M. (2020). COVID-19 Fight Enlists Digital Technology: Contact Tracing Apps. Engineering.

[37] Meier Y., Meinert J., Krämer N.C. (2021). Investigating factors that affect the adoption of COVID-19 contact-tracing apps: A privacy calculus perspective. Technol. Mind Behav..

[38] Park S., Choi G.J., Ko H. (2020). Information technology-based tracing strategy in response to COVID-19 in South Korea-privacy controversies. JAMA J. Am. Med. Assoc..

[39] Elkhodr M., Mubin O., Iftikhar Z., Masood M., Alsinglawi B., Shahid S., Alnajjar F. (2021). Technology, privacy, and user opinions of COVID-19 mobile apps for contact tracing: Systematic search and content analysis. J. Med. Internet Res..

[40] Madden M., Lenhart A., Cortesi S., Gasser U., Duggan M., Smith A., Beaton M. (2022). Teens, Social Media, and Privacy. Pew Res. Cent. Internet Am. Life Proj..

[41] Munzert S., Selb P., Gohdes A., Stoetzer L.F., Lowe W. (2021). Tracking and promoting the usage of a COVID-19 contact tracing app. Nat. Hum. Behav..

[42] Frimpong J.A., Helleringer S. (2021). Strategies to increase downloads of COVID–19 exposure notification apps: A discrete choice experiment. PLoS ONE.

[43] Fast V., Schnurr D. (2021). Incentivising the Adoption of COVID-19 Contact-Tracing Apps: A Randomised Controlled Online Experiment on the German Corona-Warn-App. Proceedings of the 2021 on Computers and People Research Conference.

[44] Albouy-Llaty M., Martin C., Benamouzig D., Bothorel E., Munier G., Simonin C., Guéant J.L., Rusch E. (2021). Positioning digital tracing applications in the management of the COVID-19 pandemic in France. J. Med. Internet Res..

