# Supplementary material for: What Went Wrong with the IMMUNI Contact-Tracing App in Italy? A Cross-Sectional Survey on the Attitudes and Experiences among Healthcare University Students
Source: Life (Basel). 2022 Jun 10;12(6):871. doi: 10.3390/life12060871 (PMC9225335; doi:10.3390/life12060871)
Supplement: Supplementary file 1 [file life-12-00871-s001.zip › life-1751116-supplementary.pdf]

Section S1 – Sociodemographic characteristics

1.1 Age: ________

1.2 Gender:
- o Male
- o Female
- o Other
- o I prefer not to declare

1.3 Field of study:
- o Nursing science
- o Physiotherapy

1.4 Year of study:
- o 1
- o 2
- o 3
- o Outside prescribed course

1.5 Nationality:
- o Italy
- o France
- o Germany
- o Other *(please specify):* _________________

1.6 If you are an Italian citizen, please write your Italian Region:________

1.7 With the financial resources at your disposal, how well do you get to the end of the month?
- o I have many difficulties
- o I have some difficulties
- o Managing well enough
- o Managing very well

1.8 What is your main source of health information?
- o Mass media
- o Social network
- o Web
- o Other *(please specify):* _________________
- o None

1.9 How often do you need to have someone help when you read instructions, pamphlets, or other written material from your doctor or pharmacy?
- o Never
- o Rarely
- o Sometimes
- o Often
- o All the time

1.10 Do you suffer from any chronic condition?
- o None
- o Asthma
- o Diabetes
- o Cancer
- o Autoimmune disease
- o Other *(please specify):* _________________

1.11 Have you ever been infected with SARS-CoV-2?
- o No
- o Yes, I have been asymptomatic
- o Yes, I have had mild symptoms
- o Yes, I have had moderate symptoms
- o Yes, I have had severe symptoms

Section S2 - COVID-19 perception and attitudes

2.1 Express your perceptions, attitudes, and feelings towards the pandemic
.
Give a rating from 1= very low or not at all to 5= very high or extremely.

	1	2	3	4	5
Fear of getting the SARS-CoV-2 infection					
Fear of infecting others					
Concern about COVID-19 emergency					
Depression					
Anxiety					
Anger					

2.2 Self-report your adherence to precautionary measures and agreement with the following statements.

For each option bar the appropriate box, from 1= not at all to 5= extremely.

	1	2	3	4	5
How much do you comply with social distancing?					
How much do you comply with the use of masks?					
I trust the response of the institutions to the emergency					
I believe in the "lab-leak theory" of the origin of COVID-19					

2.3 Has anyone ever advised you to download and use IMMUNI?
- o No
- o Yes, one of my relatives
- o Yes, one of my friends
- o Yes, my GP/another doctor
- o Other *(please specify):* _________________

2.4 Have you downloaded IMMUNI?
- o Yes, immediately after its launch (June-August 2020)
- o Yes, after last summer (September-November 2020)
- o Yes, last winter (December 2020-February 2021)
- o No

2.5 Are you still using IMMUNI on your phone?
- o Yes
- o No
- o Not applicable

Section S3A – For students who downloaded IMMUNI

3A.1 What is the main reason why you downloaded IMMUNI App?
- o Sense of duty
- o Respect for others
- o Fear of getting the infection
- o Curiosity
- o Other *(please specify):* _________________

3A.2 What is your opinion on the following aspects of IMMUNI App?

Tick the box that corresponds to your judgment, from 1 = very poor to 5 = excellent.

	1	2	3	4	5
Privacy					
Ease to use					
Usefulness					
Intuitiveness					

3A.3 Have you ever received a contact notification?
- o Yes
- o No

3A.4 What did you do after receiving the notification?
- o Not applicable
- o I received and followed the advice provided by the app
- o I received the advice, but I did not do anything

3A.5 Have you ever notified your positivity to SARS-CoV-2 through the App?
- o Not applicable
- o No, I was not able to
- o Yes, I was given the CUN, and I entered the requested data on the app
- o Yes, I provided the CUN to the healthcare professional who contacted me for contact-tracing purposes

3A.6 How do you judge the notification process?
- o Very lacking
- o Lacking
- o Good
- o Very good
- o Not applicable

3A.7 What challenges did you face during the notification process?
- o Not applicable
- o I was unable to get the CUN
- o I was unable to enter the CUN in the app even after calling the IMMUNI call center
- o I was unable to enter the CUN in the app and I did not know that I could call the IMMUNI call center
- o I did not have any difficulty
- o Other *(please specify):* _________________

You have completed the questionnaire. Thank you for participating!

<u>Section S3B – For students who did not download IMMUNI</u>

3B.1 What is the main reason why you did not download IMMUNI App?

- o I do not think it is useful
- o I did not know I had to download the app
- o Technical problems (e.g., no smartphone, operating system incompatibility, battery problems, insufficient storage on the phone, etc.)
- o I do not trust data management (privacy issue)
- o I have heard of negative personal experiences
- o Other *(please specify):* _________________

3B.2 Indicate how much these measures would incentivize your app uptake.

For each option, bar the appropriate box from 1=not at all to 5=definitely:

	1	2	3	4	5
Information on how usage can impact transmission dynamics					
Information on the app's uptake among the population					
Making the app download mandatory					
Opportunity to give feedback on the technical aspects of the app					
Information about personal data collection and management					
Economic reward					

You have completed the questionnaire. Thank you for participating!
